# *Rickettsia felis* Infections and Comorbid Conditions, Laos, 2003-2011

**DOI:** 10.3201/eid2008.131308

**Published:** 2014-08

**Authors:** Sabine Dittrich, Koukeo Phommasone, Tippawan Anantatat, Phonepasith Panyanivong, GA1/4nther Slesak, Stuart D. Blacksell, Audrey Dubot-PA(c)rA"s, JosA(c)e Castonguay-Vanier, John Stenos, Paul N. Newton, Daniel H. Paris

**Affiliations:** Lao-Oxford-Mahosot Hospital-Wellcome Trust Research Unit, Mahosot Hospital, Vientiane, Laos (S. Dittrich, K. Phommasone, P. Panyanivong, S.D. Blacksell, A. Dubot-PA(c)rA"s, J. Castonguay-Vanier, P.N. Newton, D.H. Paris);; Nuffield Department of Medicine, University of Oxford, Oxford, UK (S. Dittrich, S.D. Blacksell, A. Dubot-PA(c)rA"s, J. Castonguay-Vanier, P.N. Newton, D.H. Paris);; Faculty of Tropical Medicine, Mahidol University, Bangkok, Thailand (T. Anantatat, S.D. Blacksell, D.H. Paris);; Service Fraternel d�?(tm)Entraide�?"Laos Medical Projects, Luang Namtha, Laos (G. Slesak);; Tropenklinik Paul-Lechler-Krankenhaus, TA1/4bingen, Germany (G. Slesak);; Aix Marseille University, Medical University, Marseille, France (A. Dubot-PA(c)rA"s);; The Geelong Hospital, Geelong, Victoria, Australia (J. Stenos)

**Keywords:** *Rickettsia felis*, typhus, fleaborne, vectorborne, Lao PDR, Laos, co-infections, immunocompromised, tropical rickettsiosis

**To the Editor:** Fleaborne disease is highly prevalent in Laos, mainly attributed to murine typhus (*Rickettsia typhi* infection), transmitted by *Xenopsylla cheopis* fleas, but data on other fleaborne diseases are limited (*1*). We screened blood and cerebrospinal fluid (CSF) from participants in 2 large prospective studies in Laos for *Rickettsia* spp. using a genus-specific 17-kDa-based *Rickettsia* real-time quantitative PCR assay, and positive results were confirmed by DNA sequencing (*2**,**3*). In samples from >2,500 patients (2,540 blood and 1,112 CSF), we detected 3 cases of sequence-confirmed *R. felis* infections.

A 50-year-old man, an official in Vientiane City, was admitted to a hospital with fever and headache in October, 2008. HIV infection and cryptococcal meningitis were diagnosed. Treatment with intravenous amphotericin B, then oral fluconazole, was successful; antiretroviral treatment was initiated 1 month after diagnosis. Among a panel of diagnostic PCRs, the CSF sample specimen tested positive for genus-specific 17-kDa-*Rickettsia* quantitative PCR, but was negative for *Orentia tsutsugamushi* and *R. typhi*. DNA sequencing of 434 bp of the 17-kDa gene (Macrogen, Seoul, South Korea) revealed a 100% homology to the *R. felis* URRWXCal2 strain (Table).

**Table:** Clinical and laboratory findings of 3 patients with *Rickettsia felis* infections, Laos*

Patient characteristics	Signs and symptoms	Molecular findings	Serologic findings	Other laboratory findings
Male, 50 y, Vientiane City, central Laos	Fever, severe headache A- 7 d; contact with cats and dogs 14 d before admission; HIV/AIDS (CD4-count: 34 cell/I1/4L)	qPCR: *Orientia tsutsugamushi* (CSF, blood): negative; *Rickettsia* spp. (CSF): positive; *Rickettsia* spp. (blood): negative; *R. typhi* (CSF, blood): negative �?"Conventional PCR and sequencing (CSF):*Rickettsia* spp. 17 kDa; GenBank accession no: KF489454	Scrub/murine typhus: IgM/IgG static titers (<1:100; negative)�?"Spotted fever group: IgM/IgG static titers (1:200; negative) �?"*R. felis:* IgM/IgG static titers (<1:128; negative)	Increased intracranial pressure (>40 cm H_2_0)�?"CSF: clear �?"CSF cellularity: 5 leukocytes/I1/4L (100% lymphocytes) �?"CSF glucose: 1.1 mmol/L; CSF/blood glucose ratio: 1:5 �?"CSF protein: 80 mg/L; �?"CSF *Cryptococcus* culture: positive/serotyping (PCR/RFLP) *C. neoformans* var. *grubii* �?"HIV rapid tests;�? positive
Female, 39 y, Luang Namtha, northern Laos	Fever A- 7 d; diabetes mellitus, treated with glibenclamide; HIV status: unknown	qPCR: *O. tsutsugamushi* (eschar, blood): positive; *Rickettsia* spp. (eschar): positive; *R. typhi* (eschar): negative �?"Conventional PCR and sequencing (eschar): *Rickettsia* spp. 17kDa and *sca4;* GenBank accession no: KF489455, KF489457	Scrub typhus: dynamic IgM/IgG 4-fold rise (1:3,200/1:12,800)�?"Murine typhus: IgM/IgG static titers (<1:100; negative)�?"Spotted fever group: IgM/IgG static titers (1:200; negative); �?"*R. felis:* IgM/IgG static titers (<1:128; negative)	None
Male, 13 y, Salavan, southern Laos	Fever A- 7 d, contact with cat, rat, and fleas 14 d before admission; HIV status: unknown	qPCR: *O. tsutsugamushi* (blood): negative; *Rickettsia* spp. (blood): positive *R. typhi* (blood): negative �?"Conventional PCR and sequencing (blood): *Rickettsia* spp. 17 kDa GenBank accession no: KF489456	Data not available	Malaria microscopy: *P. falciparum;* �?"Malaria rapid test: *P. falciparum* ICT Malaria Combo Cassette Test�?�: *P. falciparum* �?"PCR: dengue fever positive; reverse transcription qPCR: positive�?"Dengue genotyping PCR: serotype 4

*qPCR, quantitative PCR; CSF, cerebrospinal fluid; leukocyte; RFLP, restriction fragment length polymorphism; ICT, immunochromatographic test.�?"�? Uni-GoldTM HIV, Trinity Biotech, Ireland; Alere Determine HIV-1/2 Ag/Ab Combo, Alere Medical, Japan.�?"�?�ICT Diagnostics, Cape Town, South Africa.

*R. felis* positivity in CSF is rare; 4 have been reported (*3*). The combined findings of *R. felis* infection and severe immunodeficiency in this patient led to a reevaluation of the 2 reported *R. felis* infections in Laos (*2*). Before this study, *R. felis* DNA or culture had not been handled in our facility. The interval between processing positive samples, dedicated separate areas for samples before and after PCR, and the low positivity rate make DNA contamination highly unlikely.

A 39-year-old housewife from Luang Namtha in northern Laos had a history of diabetes mellitus, which had been treated with glibenclamide. On arrival at the hospital in November, 2008, she had fever, headache, myalgia, and an eschar. She was empirically treated with doxycycline (Table). An eschar biopsy specimen was PCR-positive for *Rickettsia* spp. and *O. tsutsugamushi*; PCR of buffy coat detected *O. tsutsugamushi* DNA only (*2*). Molecular characterization included 17-kDa�?" and *sca4*- gene sequencing, which both revealed amplicons of 100% identity to the *R. felis-*URRWXCal2 strain. Serologic evidence for *O. tsutsugamushi* infection (scrub typhus) included a 4-fold rise in IgM and IgG titers, and IgM and IgG titers against typhus group rickettsiae, spotted fever group rickettsiae, and *R. felis* (isolate B377 in XTC-2 cells, Australian Rickettsial Reference Laboratory) were negative in admission and convalescent-phase samples (6-day interval) (Table).

A 13-year-old boy from Salavan, in southern Laos, had fever, headache, and nonspecific symptoms in July, 2009. *P. falciparum* malaria and dengue were diagnosed, both confirmed by PCR (Table). PCR results for the buffy coat specimen were positive for the 17-kDa gene; subsequent sequencing confirmed *R*. *felis* with 100% identity to the URRWXCal2 strain. The fever resolved after treatment with antimalarial drugs and ceftriaxone; neither would be expected to be efficacious for *R. felis* infection.

These data suggest that *R. felis* occurs in Laos, and is possibly emerging, but whether it results in clinical disease or commonly causes subclinical infection is unknown. The screened cohorts of consecutively enrolled patients with febrile illnesses across 3 diverse geographic regions are representative of etiologic agents of fever across Laos. PCR has previously been used for detection of *R. felis* and resulted in the discovery of a new *R. felis*-like organism in fleas in Kenya, Candidatus *Rickettsia asemboensis* (*4*). Reports from Southeast Asia suggest that *R. felis* is not a common cause of febrile illness (*1**,**2*), which contrasts with findings in Kenya, where *R. felis* was found in �%^7% of febrile patients (*4*,*5*), and also in �%^3% of afebrile patients (*5*).

The high *R. felis* carriage rate in fleas found in Laos (77% overall; 53% in *Ctenocephalides felis felis*, 89% in *C. f. orientis*) contrasts strongly with the apparent low incidence of *R. felis* human infections (*6*). Among febrile hospitalized patients in Vientiane, 1 case of *R. felis* infection was serologically diagnosed by using species-specific cross-absorption (*1*). Seroprevalence studies in the region could elucidate exposure to this pathogen and unmask subclinical infections missed in fever etiology studies.

The 3 patients from Laos described herein had comorbidities associated with variable degrees of immunodeficiency (HIV infection and malaria with cellular and humoral deficiencies, diabetes with functional neutrophil/macrophage impairment) (*7**,**8*). *R. felis* infections have not been associated with immunosuppression, but few investigations of this possible association have been published. Of the 3 patients, the woman and the boy had other vectorborne infections: scrub typhus, transmitted by *Leptotrombidium* mites, and *P. falciparum* malaria and dengue, transmitted by *Anopheles* and *Aedes* mosquitoes, respectively. Recent reports described *R. felis* within a great diversity of vectors, including mites in South Korea and *Anopheles* and *Aedes* mosquitoes in Africa (*9**,**10*).

More work is needed on the role of non-flea vectors in transmission of *R. felis* and the consequences this may have in terms of mixed infections of diverse vectorborne pathogens. The rare detection of *R. felis* in patients, combined with high flea carriage rates, unusual signs and symptoms linked to immunodeficiencies or multiple infections, and reports from Africa describing *R. felis* in asymptomatic patients, underscore the need for further investigations into the organism�?(tm)s natural history and its uncertain role as a pathogen.
